# Supplementary figures and images for: Sensor-Based Gait Retraining Lowers Knee Adduction Moment and Improves Symptoms in Patients with Knee Osteoarthritis: A Randomized Controlled Trial
Source: Sensors (Basel). 2021 Aug 18;21(16):5596. doi: 10.3390/s21165596 (PMC8402273; doi:10.3390/s21165596)

## Gait retraining group

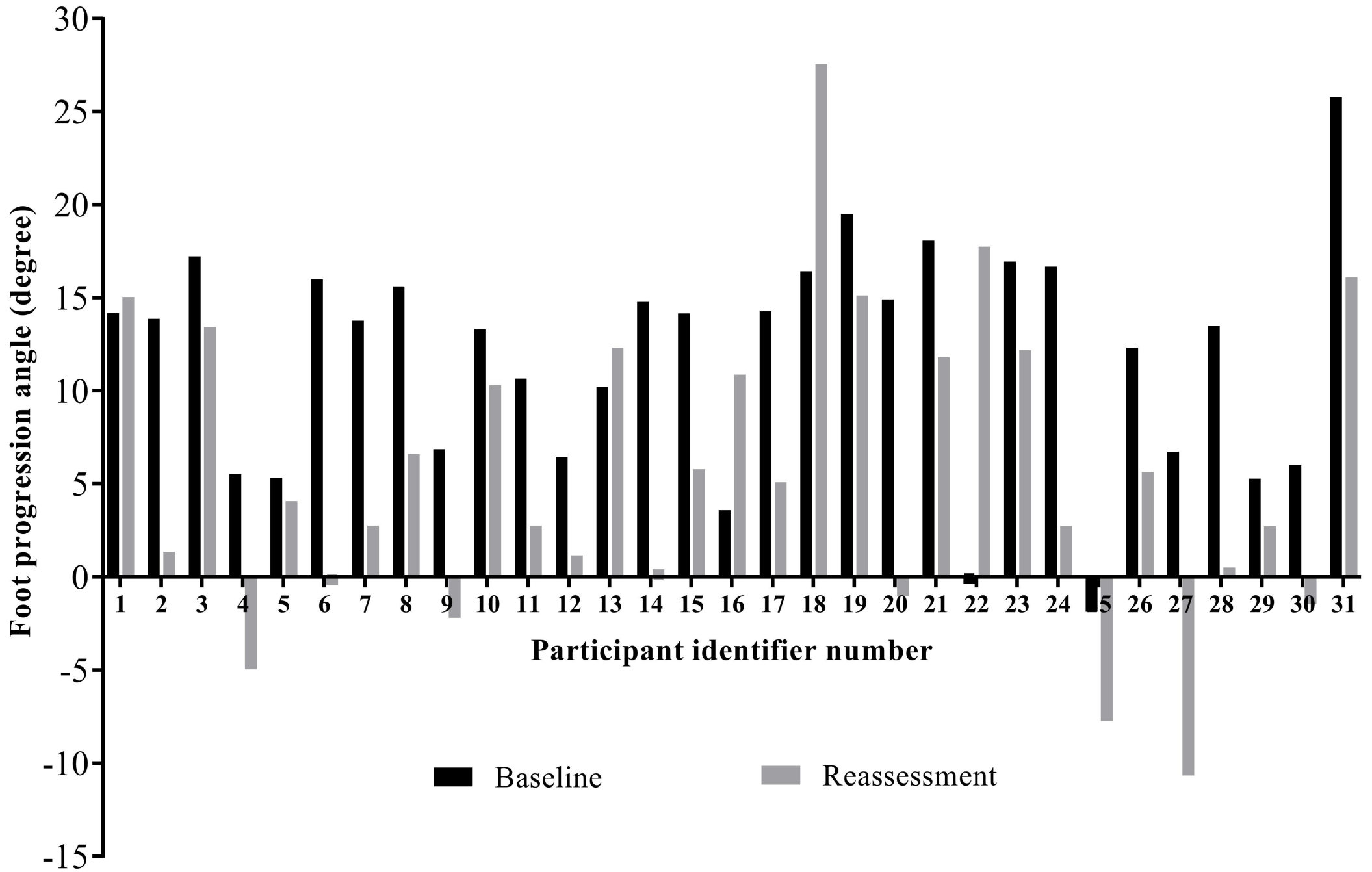

Supplement: Supplementary file 1 [file sensors-21-05596-s001.zip › sensors-1296471-supplementary.pdf]
